# A Retrospective Study Assessing the Effect of Diabetes on Mortality in Patients With COVID-19 at a Teaching Hospital in the United Kingdom

**DOI:** 10.7759/cureus.13902

**Published:** 2021-03-15

**Authors:** Fahad W Ahmed, Omar Z Kirresh, Alyss V Robinson, M S Majeed, Dominique Rouse, Rumaisa Banatwalla, Sathish Parthasarathy, Catherine Sargent, Clare Castledine, Ali J Chakera

**Affiliations:** 1 Diabetes and Endocrinology, Brighton and Sussex University Hospitals NHS Trust, Brighton, GBR; 2 Diabetes and Endocrinology, Western Sussex Hospital NHS Foundation Trust, Worthing, GBR; 3 Infectious Disease, Brighton and Sussex University Hospitals NHS Trust, Brighton, GBR; 4 Medicine, Brighton and Sussex Medical School, Brighton, GBR; 5 Diabetes and Endocrinology, Brighton and Sussex County Hospitals NHS Trust, Brighton, GBR

**Keywords:** covid-19, diabetes mellitus, united kingdom, mortality, coronavirus disease 2019

## Abstract

Aim

The aim of the study was to compare the clinical characteristics and outcomes (mortality, intensive care admission, mechanical ventilation, and length of stay, LoS) of patients with and without diabetes with confirmed COVID-19.

Methods

This retrospective study evaluated clinical and laboratory variables in adult inpatients from Brighton and Sussex University Hospitals NHS Trust with laboratory-confirmed COVID-19 between March 10, 2020, and June 30, 2020. Univariate and multivariate analyses were performed to compare the outcomes of patients with and without diabetes.

Results

Over 457 patients were included in this study (140 with diabetes and 317 without diabetes), of which 143 (31.9%) died. The median age was 80 years and were predominantly males (59.1%). Baseline characteristics at the time of COVID-19 diagnosis demonstrated that the patients with diabetes were younger than those without diabetes (p=0.008). Mortality increased with age. There was no difference in adverse outcomes in those with and without diabetes. However, subgroup analysis of patients aged ≤60 years demonstrated a significantly increased mortality in those with diabetes (p=0.016). Patients with diabetes had an increased length-of-stay compared to those without diabetes, which was more evident in those aged ≤60 years.

Conclusion

Age is the most important predictor of mortality. Patients with diabetes did not have increased mortality from COVID-19, which is likely due to their younger age in our cohort. More patients with diabetes stayed in the hospital longer than seven days than those without diabetes.

## Introduction

The world is presently undergoing a significant health crisis due to coronavirus disease 2019 (COVID-19). COVID-19 is caused by severe acute respiratory syndrome coronavirus 2 (SARS-CoV-2). The World Health Organisation declared COVID-19 as a pandemic on March 11, 2020 [1]. Two days later, Europe was declared as an epicenter of the pandemic [2]. On October 4, 2020, there were approximately 35 million cases of COVID-19 resulting in over one million deaths worldwide [3].

Several studies have demonstrated a higher proportion of patients with diabetes are admitted to hospital due to COVID-19 [4,5]. Data from the United States identified that out of 1122 patients hospitalized due to COVID-19, 40.2% (n=451) had diabetes [4]. One retrospective study from the United Kingdom (UK) observed that 37.5% of patients admitted with COVID-19 had co-existing diabetes [5]. Multiple retrospective studies and systematic reviews have demonstrated that diabetes and intercurrent COVID-19 are associated with adverse outcomes, including increased mortality [4,6-8].

This study reports the outcomes of patients admitted with COVID-19 at Brighton Sussex University Hospitals NHS Trust (BSUH), UK. This study evaluates the impact of diabetes on mortality, intensive care admission, mechanical ventilation, and length-of-stay (LoS) of hospitalized patients with COVID-19.

## Materials and methods

Study design

This retrospective cross-sectional observational study was conducted in BSUH, which comprises of two hospitals: Royal Sussex County Hospital and Princess Royal Hospital. BSUH serves a population of approximately 500,000 people. All adult patients (≥18-years) hospitalized between March 10, 2020, and June 30, 2020, with laboratory-confirmed COVID-19 [a reverse-transcriptase-polymerase-chain-reaction (RT-PCR) SARS-CoV-2 assay of a nasopharyngeal swab], were included in the analysis. Patients were excluded from the analysis if they were discharged from the emergency department, transferred to another center (predominantly occurring in the early stages of the COVID-19 pandemic in the UK), or there was insufficient clinical information. Ethics approval was not required as the data were collected anonymously as part of routine healthcare delivery and service improvement analysis.

Data collection

Clinical and laboratory data were extracted from electronic hospital records. The clinical information collected were age, biological sex, ethnicity, socio-economic status, pre-existing diabetes or no pre-existing diabetes, type of diabetes (type 1 diabetes mellitus or type 2 diabetes mellitus), co-morbidities [chronic obstructive pulmonary disease (COPD), asthma, heart disease, hypertension, malignancy], length-of-stay, admission to intensive care unit (ITU), requiring mechanical ventilation and mortality. The laboratory parameters collected were glycated hemoglobin (HbA1c), estimated glomerular filtration rate (eGFR), and admission venous blood gas (VBG) glucose. Ward-based capillary glucose readings were reviewed to evaluate inpatient glycaemic control for patients with diabetes.

Diabetes was defined as an HbA1c ≥6.5% (≥48 mmol/mol) on admission or an established diagnosis of diabetes prior to hospitalization. Socio-economic status was based on English Indices of Multiple Deprivation for the postcode (2019) [9]. Index of multiple deprivations provides an official measure of deprivation of a local area across England and is based on different factors, e.g., income, employment, education, health and disability, crime, living environment, and housing barriers [9]. The area with an index of multiple deprivation score of 1 is the most deprived area whereas the area with an index of multiple deprivations of 10 is the least deprived area [9]. Diabetic ketoacidosis (DKA) and hyperosmolar hyperglycaemic state (HHS) were defined as per the Joint British Diabetes Societies (JBDS) guidance [10,11]. Hyperglycaemia on admission was described as a blood glucose level >11 mmol/l at the time of admission on VBG. Hyperglycaemia during admission was analyzed in two ways (1) average blood glucose level >11 mmol/l during admission (capillary blood glucose) and (2) two blood glucose readings >11 mmol/l within a 24-hour period during admission. Hypoglycemia at presentation was defined as blood glucose <4 mmol/L.

Statistical analysis

Categorical variables were reported as percentages. Continuous variables were reported as a median and inter-quartile range (IQR) as they were not normally distributed. Variables were compared between patients with diabetes and without diabetes. Furthermore, we compared ITU admissions, mechanical ventilation, and length of stay (LoS) between patients based on diabetes status and age ≤60 and >60 years. We also compared age between ethnic groups.

In patients with diabetes, individual variables were assessed according to mortality outcomes. For continuous variables, the Mann-Whitney U test was used for non-normally distributed variables, whereas Pearson’s chi-squared test (χ²) was used for categorical variables.

Logistic regression analysis was undertaken to calculate the odds for all-cause 28-day mortality in both the whole cohort and in the diabetes subset. Variables with a p-value <0.05 in the univariate analysis and those thought to be clinically related to the associations examined were included in the multivariate analysis.

All-cause mortality was calculated as time, in days, between the first positive RT-PCR SARS-CoV-2 swab date and the date of death within 28 days of a positive COVID-19 test. Follow-up is defined from the date of positive RT-PCR SARS-CoV-2 swab to death or within 28 days of a positive COVID-19 test (whichever comes first). This is based on the Public Health England criteria used to calculate COVID-19 daily death [12]. Survival analysis was undertaken using the Kaplan-Meier survival curve based on (1) diabetes status (all ages included), and (2) diabetes and age ≤60 years and >60 years. A p-value of <0.05 was considered statistically significant. The analysis was performed with SPSS software (version 23.0, IBM Corp., Armonk, NY).

## Results

Overall results

**Figure 1:**
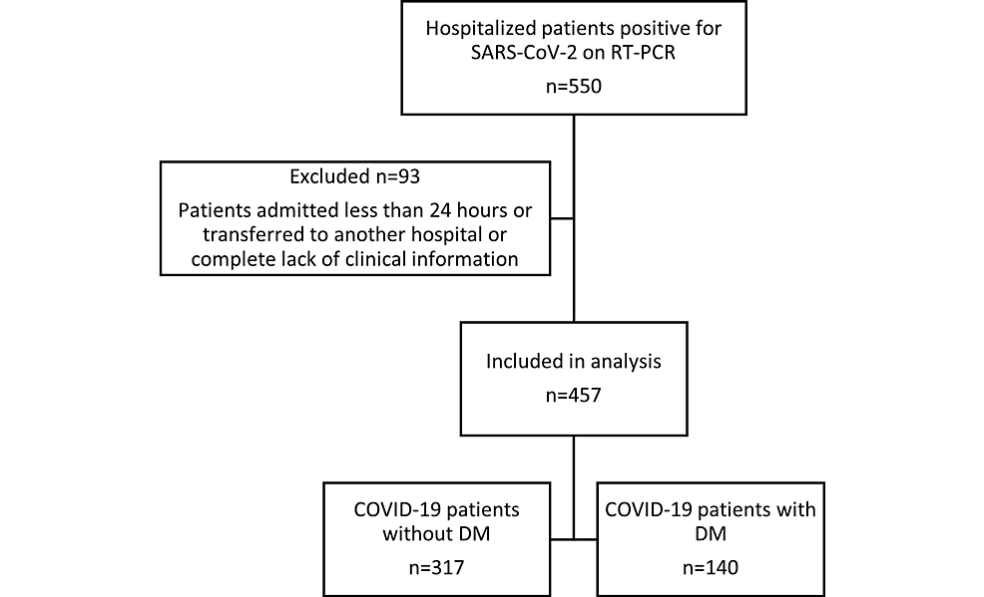
Flow diagram of patients included in the study. COVID-19: coronavirus, SARS-COV-2: severe acute respiratory syndrome coronavirus-2, RT-PCR: real-time reverse transcription-polymerase chain reaction, n: number.

During the study period, 550 patients were identified to have tested positive for SARS-CoV-2. After pre-protocol exclusions, 457 patients were included in the analysis (Figure 1). Baseline characteristics and laboratory findings of all patients and those with and without diabetes are summarised in Table 1. There was a greater proportion of males (59.1%) than females, and the median age was 80 years. The median LoS was 14 days (6-26). Out of 457 patients, 140 (30.6%) had diabetes and 143 (31.9%) patients with COVID-19 died. Patients from a Black, Asian, and minority ethnic (BAME) background were significantly younger than patients from a White background [median (IQR); BAME: 60.5 (46-72) years vs 81 (69.50-87) years: p<0.0001].

**Table 1:** Baseline characteristics and outcomes for all hospitalized COVID-19 patients with and without diabetes. n: number of patients, %: percentage, BAME: Black, Asian and Minority Ethnic, COPD: chronic obstructive pulmonary disease, eGFR: estimated glomerular filtration rate, ITU: intensive care, IQR: interquartile range, LoS: length-of-stay.

	n	All	n	Non-diabetes	n	Diabetes	p-Value
Demographics							
Male sex (n, %)	457	270 (59.1%)	317	185 (58.4%)	140	85 (60.7%)	0.68
Age (median, IQR)	457	80 (67-87)	317	81 (68-87)	140	76 (64-85)	0.008
Ethnicity (n, %)	457		317		140		
White		389 (85.1%)		279 (88%)		110 (78.6%)	0.001
BAME		46 (10.1%)		21 (6.6%)		25 (17.9%)	
Unknown		22 (4.8%)		17 (5.4%)		5 (3.6%)	
Co-morbidities (n, %)							
Asthma	449	45 (9.8%)	311	25 (8%)	138	20 (14.5%)	0.04
COPD	449	59 (12.9%)	311	34 (10.9%)	138	25 (18.1%)	<0.05
Heart disease	449	172 (37.6%)	311	105 (33.8%)	138	67 (48.6%)	0.003
Hypertension	449	187 (40.9%)	311	109 (35%)	138	78 (56.5%)	<0.0001
Cancer	448	98 (21.4%)	311	66 (21.2%)	137	32 (23.4%)	0.62
Laboratory findings							
HbA1c (median, IQR)	358	41 (37-49)	244	38 (35-41)	134	55.5 (46-70.25)	0.0001
Venous glucose (median, IQR)	413	6.7 (5.8-8.75)	288	6.35 (5.57-7.2)	125	9.2 (6.95-12.2)	<0.0001
eGFR (n, %)	456						
≥60		255 (55.9%)		192 (60.6%)		63 (45.3%)	0.03
<60		201 (44.1%)		125 (39.4%)		76 (54.7%)	
Index of multiple deprivation decile (n, %)	457		317		140		
1-3		78 (17.1%)		53 (16.7%)		25 (17.9%)	0.76
4-7		191 (41.8%)		130 (41%)		61 (43.6%)	
8-10		188 (41.1%)		134 (42.3%)		54 (38.6%)	
Outcomes (n, %)							
Death	457	143 (31.9%)	317	101 (31.9%)	140	42 (30.0%)	0.74
ITU admission	457	52 (11.4%)	317	34 (10.7%)	140	18 (12.9%)	0.53
Mechanical ventilation	457	18 (3.9%)	317	12 (3.8%)	140	6 (4.3%)	0.80
LoS (median, IQR)	457	14 (6-26)	317	13 (5-25)	140	14.5 (8.25-26.75)	0.057
LoS (n, %)							
<7 days		127 (27.8%)		100 (31.6%)		27 (19.3%)	0.019
7-14 days		116 (25.4%)		73 (23%)		43 (30.7%)	
>14 days		214 (46.8%)		144 (45.4%)		70 (50%)	

Baseline characteristics in patients with diabetes and without diabetes

Patients with diabetes were significantly younger and with more co-morbidities than those without diabetes (Table 1). There was a significantly greater proportion of BAME backgrounds in those with diabetes (17.9%) compared to those without diabetes (6.6%; p<0.001). Patients from a BAME background in our cohort were significantly younger than those from a White background in both diabetes and non-diabetes groups [median (IQR); diabetes: BAME - 66 (49-79) years vs White - 78 (67-85.25) years: p=0.017; non-diabetes: BAME - 55 (43.50-67.50) years vs White - 81 (59.50-77) years: p<0.0001].

There was no significant difference in mortality outcomes and median LoS in patients with and without diabetes. However, the distribution of LoS demonstrated more patients without diabetes were discharged within a week of admission.

Admission blood glucose was significantly higher in those with diabetes (9.2 mmol/L) compared to those without diabetes (6.35 mmol/L; p<0.0001). There was a significant difference in the number of patients with an eGFR<60 in patients with diabetes than those without diabetes (54.7% versus 39.4%, p=0.03).

The number of patients requiring ITU admission and mechanical ventilation and length-of-stay outcomes of COVID-19 patients based on age (≤60 years compared to >60 years) and diabetes status are provided in Table 2. There was no significant difference in age between diabetes and non-diabetes groups. In patients aged ≤60 years, those with diabetes had a significantly longer LoS than those without diabetes [median (IQR); Diabetes: 11 (8-16.75) days vs non-diabetes: 5 (3-12.25) days: p=0.002]; 53.8% with diabetes had an LoS of 7-14 days, whilst 12.5% without diabetes had an LoS of 7-14 days. There was no significant difference between ITU admissions and mechanical ventilation in patients ≤60 years with diabetes and those without diabetes.

**Table 2:** ITU admission, mechanical ventilation, and length outcomes of COVID-19 patients based on age (≤60-years-old compared to >60-years-old) and diabetes status hospitalized at Brighton and Sussex University Hospitals. ITU: intensive care, n: number, %: percentage, LoS: length of stay.

	Non-diabetes	Diabetes	p-Value
Number of patients (n, %)			
≤60 years	48 (15.1%)	26 (18.6%)	0.41
>60 years	269 (84.9%)	114 (81.4%)	
ITU admission (n, %)			
Age ≤60 years	8 (16.7%)	7 (26.9%)	0.37
Age >60 years	26 (9.7%)	11 (9.6%)	0.99
Mechanical ventilation (n, %)	12 (3.8%)	6 (4.3%)	0.80
Age ≤60 years	3 (6.3%)	1 (3.8%)	0.99
Age >60 years	9 (3.3%)	5 (4.4%)	0.77
LoS (n, %)			
Age ≤60 years			
<7 days	31 (64.6%)	4 (15.4%)	<0.0001
7-14 days	6 (12.5%)	14 (53.8%)	
>14 days	11 (22.9%)	8 (30.8%)	
Age >60 years			
<7 days	69 (25.9%)	23 (20.2%)	0.50
7-14 days	67 (24.9%)	29 (25.4%)	
>14 days	133 (49.4%)	62 (54.4%)	

There was no significant difference in LoS [median (IQR); diabetes: 15 (9-27) days vs non-diabetes: 14 (6-26) days: p=0.24], ITU admissions, and mechanical ventilation in patients aged >60 years in those with and without diabetes.

Characteristics in COVID-19 patients with diabetes and mortality outcome

There was no significant difference in the mortality outcomes of COVID-19 patients with diabetes (Table 3) in relation to biological sex, age, ethnicity, type of diabetes, index of deprivation decile, HbA1c, control of diabetes (VBG glucose, average glucose, hyperglycemia at presentation, hyperglycaemic emergency, hypoglycemia, and the number of capillary blood glucose readings) co-morbidities, and eGFR. Average capillary blood glucose was [median (IQR) 8.6 (7.1-10.33) mmol/L].

**Table 3:** Clinical characteristics based on mortality outcomes for hospitalized patients with COVID-19 and diabetes. n: number, %: percentage, BAME: Black Asian Minority Ethnicity, LoS: length-of-stay, COPD: chronic obstructive pulmonary disease, HTN: hypertension, ITU: intensive care unit, IQR: intra-quartile, eGFR: estimated glomerular filtration rate, DKA: diabetic ketoacidosis, HHS: hyperosmolar hyperglycemic state. *≤8.5%, ^>8.5%.

	n	Not alive	n	alive	p-Value
Demographics					
Male sex (n, %)	42	28 (66.7%)	98	57 (58.2%)	0.45
Age (median, IQR)	42	78 (70-85)	98	72.5 (61.75-85.25)	0.19
Age (n, %)					
≤60 years		3 (7.1%)		23 (23.5%)	0.03
>60 years		39 (92.9%)		75 (76.5%)	
Ethnicity (n, %)	42		98		
White		35 (83.3%)		75 (76.5%)	0.66
BAME		6 (14.3%)		19 (19.4%)	
Unknown		1 (2.4%)		4 (4.1%)	
Co-morbidities (n, %)					
Asthma	40	2 (5%)	98	18 (18.4%)	0.06
COPD	40	10 (25%)	98	15 (15.3%)	0.22
Heart disease	40	20 (50%)	98	47 (48%)	0.85
Hypertension	40	21 (52.5%)	98	57 (58.2%)	0.57
Cancer	40	11 (27.5%)	97	21 (21.6%)	0.51
Glycemic parameters					
HbA1c (median, IQR)	39	57 (48-68)	95	53 (45-72)	0.51
HbA1≤69 mmol/mol* (n, %)		31 (79.5%)		68 (71.6%)	0.39
HbA1c>69mmol/mol^ (n, %)		8 (20.5%)		27 (28.4%)	
Venous glucose (median, IQR)		8.6 (6.7-12.2)		9.5 (7.2-12.4)	0.44
Hyperglycemia > 11mmol/L, two readings per day (n. %)	40	24 (61.5%)	94	53 (57%)	0.70
Average glucose, mmol/L (median, IQR)	40	8.55 (7.1-10.23)	94	8.6 (7.2-10.9)	0.57
Hyperglycemia with average reading > 11 mmol/L (n, %)	40	9 (22.5%)	94	18 (19.1%)	0.65
Point of care readings (median, IQR)	42	3.4 (2.4-4.4)	98	3.4 (1.9-4.4)	0.58
Point of care readings (n, %)	42		98		
≤2		10 (24.4%)		30 (30.6%)	0.54
>2		31 (75.6%)		68 (69.4%)	
Hyperglycemia on admission (n, %)	40	11 (27.5%)	85	28 (32.9%)	0.68
DKA or/and HHS, n (%)	42	2 (4.8%)	98	3 (3.1%)	0.64
Hypoglycemia on admission (n, %)	42	1 (2.4%)	98	1 (1%)	0.51
Laboratory findings					
eGFR (n, %)	42		97		
<60		15 (35.7%)		48 (49.5%)	0.14
≥60		27 (64.3%)		49 (50.5%)	
Index of multiple deprivation decile (n, %)	42		98		
1-3		9 (21.4%)		16 (16.3%)	0.46
4-7		20 (47.6%)		41 (41.8%)	
8-10		13 (31%)		41 (41.8%)	

Risk factors associated with mortality

Table 4 describes univariate and multivariate logistic regression analyses of the risk factors for mortality in all patients with COVID-19 hospitalized in BSUH, UK. In the multivariate analysis of all the patients with COVID-19, age >60 years and an eGFR <60 were independent risk factors associated with increased mortality.

**Table 4:** Logistic regression analysis for evaluating the risk factors for mortality in all patients with COVID-19 hospitalized in BSUH, United Kingdom. P<0.05 was considered significant. *Used for multivariate analysis, **compared to ≤60 years of age, ***include diabetes, asthma, COPD, heart disease, hypertension and cancer, ^HbA1c > 8.5%, ^^compared to type 1 diabetes, +diabetes excluded from risk factor (included Asthma, COPD, heart disease, hypertension and cancer), COPD: chronic obstructive pulmonary disease, BAME: Black Asian Minority Ethnicity, eGFR: estimated glomerular filtration rate, ND: not done.

	All participants				Diabetes	
	Univariate		Multivariate		Univariate	
	p-Value	OR; CI 95%	p-Value	OR; CI 95%	p-Value	OR; CI 95%
Age >60 years^**^	<0.0001^*^	13.64 (4.22-44.10)	0.001	12.78 (3.01-54.22)	0.032	3.99 (1.13-14.11)
Male	0.052	1.504 (0.99-2.27)	ND	ND	0.342	1.44 (0.68-3.07)
Diabetes	0.392	0.92 (0.60-1.41)	ND	ND	ND	ND
Asthma	0.059	0.47 (0.21-1.03)	ND	ND	0.06	0.23 (0.05-1.06)
COPD	0.006^*^	2.18 (1.25-3.80)	0.058	1.75 (0.98-3.12)	0.18	1.84 (0.75-4.55)
Heart disease	0.037^*^	1.55 (1.03-2.33)	0.95	1.01 (0.65-1.58)	0.83	1.09 (0.52-2.26)
Hypertension	0.732	0.93 (0.62-1.40)	ND	ND	0.54	0.80 (0.38-1.67)
Cancer	0.026^*^	1.70 (1.07-2.71)	0.16	1.42 (0.87-2.33)	0.46	1.37 (0.60-3.20)
Risk Factors (1 or more)^***^	0.273	0.91 (0.78-1.07)	ND	ND	0.23^+^	1.45 (0.79-2.64)
eGFR <60 mL/min/1.73 m^2^	<0.001^*^	2.49 (1.678-3.73)	0.001	2.06 (1.33-3.20)	0.14	1.76 (0.84-3.72)
Hyperglycemia on presentation	0.820	0.93 (0.49-1.77)	ND	ND	0.54	0.77 (0.34-1.77)
BAME	0.036^*^	0.43 (0.20-0.95)	0.24	0.55 (0.21-1.47)	0.45	0.68 (0.25-1.84)
Type 2 diabetes^^^^	ND	ND	ND	ND	0.62	0.63 (0.10-3.93)
Hyperglycemic emergencies	ND	ND	ND	ND	0.62	1.58 (0.26-9.84)
Hyperglycemia (two readings >11 mmol/L)	ND	ND	ND	ND	0.63	1.21 (0.56-2.59)
Average glucose > 11 mmol/L	ND	ND	ND	ND	0.66	1.23 (0.50-3.02)
Number of blood glucose reading per day	ND	ND	ND	ND	0.46	1.37 (0.60-3.14)
HbA1c >69^^^ mmol/mol	ND	ND	ND	ND	0.35	0.65 (0.26-1.59)

Univariate analysis (Table 4) of patients with diabetes found that age >60 was the only risk factor; therefore, multivariate analysis was not performed.

Survival analysis

**Figure 2:**
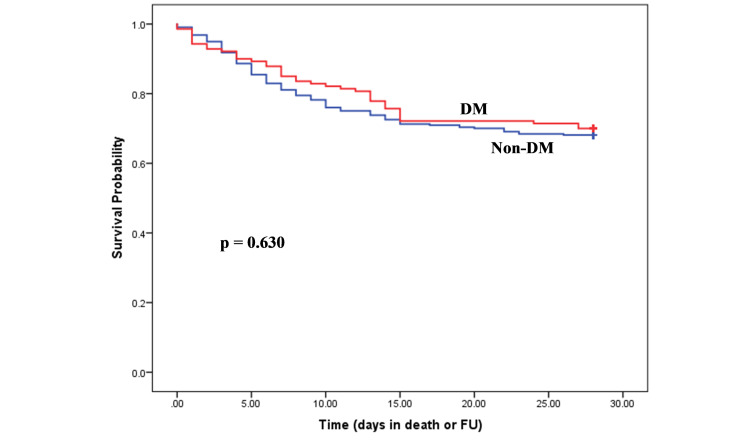
Kaplan-Meier estimates of survival for COVID-19 patients with and without diabetes mellitus.

**Figure 3:**
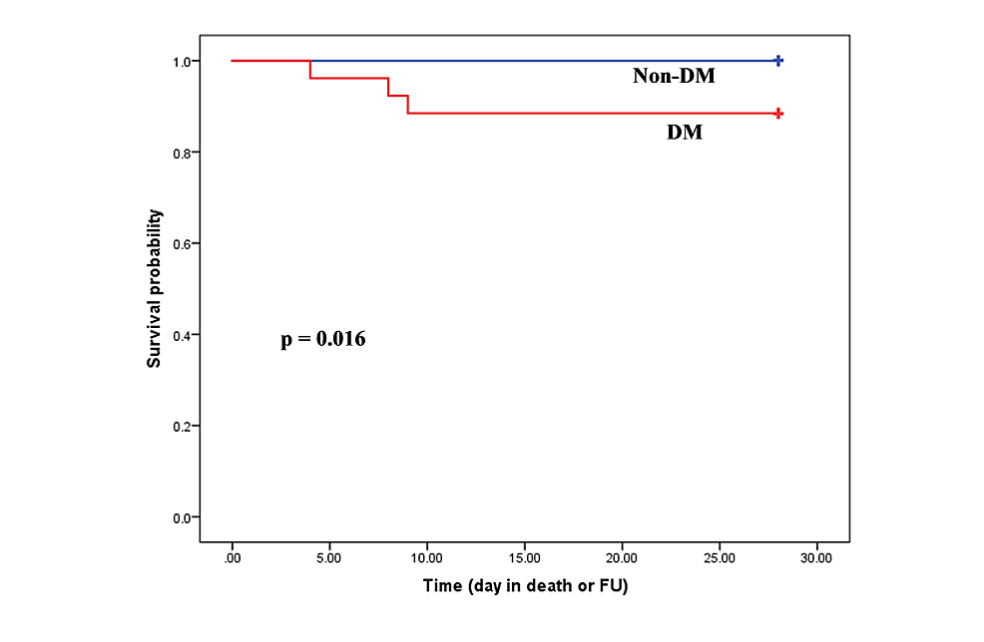
Kaplan-Meier estimates of survival for COVID-19 patients with and without diabetes mellitus aged ≤60 years.

**Figure 4:**
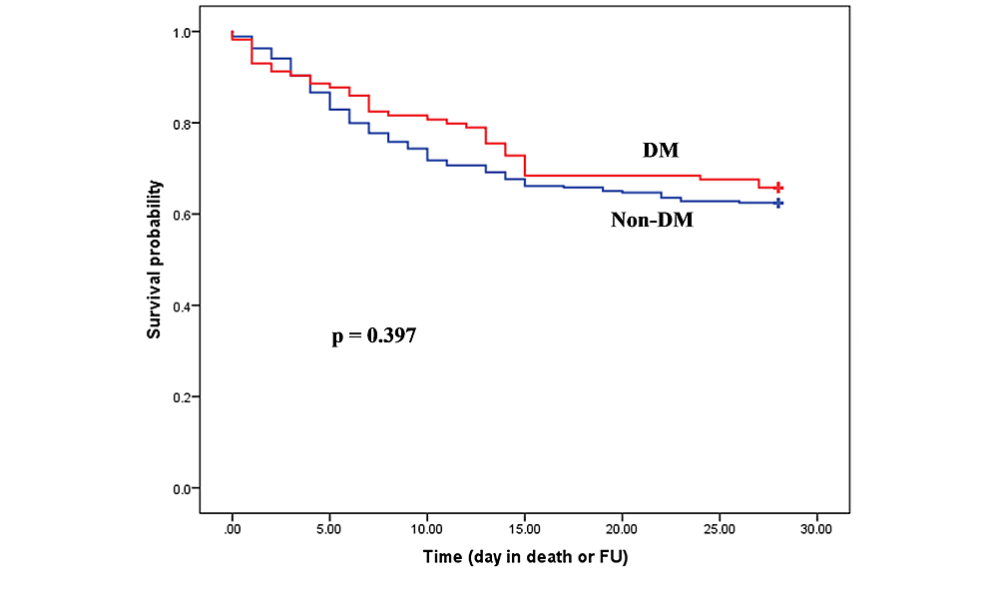
Kaplan-Meier estimates of survival for COVID-19 patients with and without diabetes mellitus aged >60 years.

Kaplan-Meier survival curves demonstrated no difference in survival in hospitalized COVID-19 patients with diabetes compared to those without diabetes (Figure 2). However, patients aged ≤60 years with diabetes (n=3) were significantly less likely (p=0.016) to survive than those without diabetes (n=0; Figure 3). Out of three, two patients were from BAME background. No significant difference was demonstrated in survival in the patients aged >60 years with or without diabetes (Figure 4).

## Discussion

This retrospective study described clinical characteristics of patients with and without diabetes, and COVID-19 admitted to a tertiary care hospital in the United Kingdom. Around a third (140 out of 457) of the patients with COVID-19 had diabetes. This study demonstrated that patients with diabetes did not have an increased risk of death, ITU admission, or mechanical ventilation. This was despite patients with diabetes having more co-morbidities and a larger proportion of BAME background. This lack of difference is likely due to the age difference between diabetes and non-diabetes groups. Subgroup analysis revealed significantly increased mortality in patients with diabetes who were aged ≤60 years compared to those without diabetes, though the numbers involved in this subgroup analysis included very few deaths.

Characteristics of all patients admitted with COVID-19

This study showed that most of the patients admitted were male (59.1%) and elderly (median age of 80 years). There were fivefold more patients aged >60 years compared to those aged ≤60 years. Furthermore, age >60 years was associated with increased mortality. These findings are consistent with current worldwide evidence [13], including the UK [5,14]. A UK study confirmed a 20-fold increased risk of COVID-19-related mortality in peoples aged >80 years compared to those <60 years [14]. The cohort in our study was significantly older (median 80 years, mean 75 years) when compared to evidence emerging from China (60 years) [15] and Europe (70 years) [5,16]. While male sex has been associated with increased mortality from COVID-19 [14,17], we did not directly observe this in our study. However, a greater proportion of hospitalized patients were men. A UK study demonstrated that males had significantly greater mortality than females [fully adjusted HR 1.5 (1.52-1.65)] [14]. This is likely due to the difference in research methods including sample size, population type, and definition of COVID-19 related mortality.

Hypertension, followed by heart disease was the most commonly encountered co-morbidities amongst the hospitalized COVID-19 patients in this study. Neither was associated with increased mortality, admission to ITU, or mechanical ventilation which is in keeping with recent literature [13,16]. An eGFR of <60 mL/min/1.73 m^2^ at the time of COVID-19 diagnosis was found in 44.1% of the patients (patients with chronic kidney disease [CKD] and acute kidney injury were both included), and this was associated with increased mortality. This supports recently published data that CKD and reduced admission eGFR were associated with increased mortality [18,19]. Angiotensin-converting enzyme 2 (ACE2) is thought to play an important role in acute kidney injury in patients with COVID-19. ACE2 is a membrane protein expressed in the kidneys, in addition to the lungs and heart [20]. The membrane spike protein on SARS-CoV-2 is thought to bind ACE2 receptors, permitting viral entry. The SARS-CoV-2 may infiltrate renal cells via the ACE2 receptor resulting in architectural damage. Furthermore, it has been proposed that COVID-19 has a direct cytotoxic effect on the kidneys [21].

Prevalence of diabetes in patients with COVID-19

Current evidence has demonstrated patients with COVID-19 and diabetes are more likely to develop severe COVID-19 [22]. 30.6% of patients admitted to BSUH with COVID-19 had diabetes. Therefore, the prevalence of hospitalized patients with diabetes and COVID-19 was seven times greater than the local population prevalence of 4.2% in Brighton and Hove, UK [23]. Alkundi et al. similarly reported a prevalence of 37.5% [5]. However, our study had more patients with type 2 diabetes (96.4%) when compared to Alkundi et al. (87.4%) [5]. The number of patients with type 1 diabetes mellitus is too small to be representative of the population with type 1 diabetes. The current literature suggests that patients with type 1 diabetes mellitus are not at increased risk of hospitalization due to COVID-19 [24].

Outcomes of COVID-19 in patients with diabetes

Even though 29.4% of the total deaths were in patients with diabetes, there was no association with increased mortality. Patients with diabetes were significantly younger than those without diabetes, which may be why there was no difference in mortality between the two groups. Univariate analysis in the diabetes group showed that age was the most significant factor predicting mortality. However, multivariate analysis in the diabetes group could not be undertaken. There is conflicting data in the literature as to whether diabetes is associated with increased mortality in COVID-19 [5,25]. Although our results are in keeping with a single center UK-based study [5], they contrast a larger population-based study [8], which showed diabetes was associated with increased mortality. In some studies, poorer outcomes in patients with diabetes could be due to significantly older age in patients with diabetes than those without diabetes [15,25].

The proportion of patients in the ≤60 and >60 years age groups was similar in diabetes and non-diabetes groups. All patients ≤60 years old who died from COVID-19 had diabetes, although this represented only three patients. In patients ≤60 years of age with diabetes, two out of three deaths occurred in the BAME population. However, regression analysis did not show BAME background was a risk factor for increased mortality. This is likely due to younger patients with a BAME background. There was no difference in mortality in patients with and without diabetes in >60 years of age.

It is well known that diabetes is associated with longer LoS in hospitalized patients [26]. We demonstrated that overall LoS from COVID-19 in our study was not significantly longer in the diabetes group. However, subgroup analysis demonstrated significantly more patients with diabetes stayed seven days or longer than those without diabetes, which may imply an increased COVID-19 disease severity and associated complications, hence requiring an extended admission. Furthermore, we demonstrated an increased LoS in patients with diabetes who were ≤60 years. In contrast, patients aged >60 years did not show any difference in LoS. Current evidence with regards to COVID-19 and diabetes has been conflicting [15,27]. Wang et al. showed that there was no difference in LoS between patients with diabetes and those without diabetes [27]. In contrast, Chen et al. showed that patients with diabetes had a significantly longer LoS than those without diabetes [15].

COVID-19 patients with diabetes and co-morbidities

Our study showed that patients with diabetes had significantly more co-morbidities (HTN, heart disease, COPD, asthma, and renal impairment). However, these co-morbidities were not associated with increased mortality in patients with COVID-19 and diabetes. eGFR was associated with increased mortality in the overall analysis of all patients. Our data are supported by current evidence with regards to HTN, heart disease, and eGFR [13,19].

Impact of glycemia in patients with COVID-19 and diabetes

Poor glycaemic control in patients with COVID-19 has been associated with worse outcomes, including mortality [4]; however, we did not observe this within our cohort. This could be due to the differences in age between the patients with and without diabetes in our study compared to these studies. Patients with hyperglycaemic emergencies (DKA or/and HHS) did not have increased mortality. There are reports showing that patients with diabetes and COVID-19 are more likely to present with DKA and/or HHS [28]. It is known that patients with hyperglycaemic emergencies have increased mortality and morbidity [10,11]. In contrast, a recent study demonstrated that patients with COVID-19 and DKA had improved survival to those without DKA [5]. Further studies are needed to evaluate the effect of diabetic emergencies in patients with diabetes and COVID-19.

We demonstrated that the median blood glucose in patients with diabetes were acceptable. Hyperglycaemia has been reported with COVID-19 in patients with and without diabetes and is associated with worse outcomes, including increased mortality [16]. This is important in the context of the recent guidelines recommending administering dexamethasone in severe COVID-19 [29]. Therefore, an increased frequency of monitoring should be undertaken in patients with diabetes (in our study, around 28% of the patient had capillary blood glucose tested on ≤2 occasions).

Limitations

Our study has some limitations. First, due to the retrospective study design, body mass index was not available, which is an important independent risk factor for mortality in COVID-19 [30]. Second, we did not include the duration of diabetes as this was not available in most of the records. Therefore, we could not ascertain if obesity and duration of diabetes are confounding variables affecting COVID-19 mortality. Third, we defined COVID-19-related death as patients dying within 28 days of a first positive laboratory-confirmed COVID-19 test. It may mean that we have under-calculated the number of deaths. In England, 88% of the deaths reported up to August 3, 2020 occurred within 28 days of the first COVID-19 positive test. Death in patients who have tested positive for COVID-19 becomes progressively less likely to be as a result of COVID-19 as the time from the diagnosis increases. Therefore, we based the mortality on Public Health England guidance [12]. In addition, as stated above, subgroup mortality analysis aged ≤60 years included very few deaths, therefore, the result should be interpreted with caution. Our study is strengthened by only including patients who were SARS-CoV-2 PCR positive. We evaluated glycaemic control at the time of admission and during the hospital stay in patients with diabetes. Blood glucose levels at the time of admission may cause bias, particularly from reverse causality. Hyperglycemia has been associated with increasing severity of COVID-19 infection and poor prognosis for patients with or without diabetes [7]. Finally, all the data were obtained for patients during the first wave of the COVID-19 pandemic. The management of COVID-19 underwent significant changes including the use of Dexamethasone and Remdesivir. Some of the patients may have received these medications especially in the later cohort which could have influenced the outcome.

## Conclusions

In conclusion, diabetes was only associated with increased mortality in patients ≤60 years old. However, due to the small sample size, the results of the subgroup ≤60 years old should be interpreted with caution. There was no association between glycaemic control, prior to and during hospitalization, and mortality. Patients with diabetes had longer hospital stays, which may imply greater disease severity. Older age was a significant risk factor for increased mortality in COVID-19. Prospective studies of patients with diabetes hospitalized with COVID-19 are required to determine the impact of glycemic emergencies and glycemic control on outcomes. Additional studies from other regions of the UK are needed to address whether there are regional differences in risks for mortality in patients with COVID-19.
